# Hydroxylation of recombinant human collagen type I alpha 1 in transgenic maize co-expressed with a recombinant human prolyl 4-hydroxylase

**DOI:** 10.1186/1472-6750-11-69

**Published:** 2011-06-24

**Authors:** Xing Xu, Qinglei Gan, Richard C Clough, Kameshwari M Pappu, John A Howard, Julio A Baez, Kan Wang

**Affiliations:** 1 Interdepartmental Plant Biology Major, Iowa State University, Ames, IA 50011-1010, USA; 2 Department of Agronomy, Iowa State University, Ames, IA 50011-1010, USA; 3 Formerly of ProdiGene, Inc., USA; 4 Current address: Texas Engineering Experiment Station, Food Protein R&D Center, College Station, Texas 77843, USA; 5 Current address: University of South Florida, Tampa, FL 33620, USA; 6 Applied Biotechnology Institute, San Luis Obispo, CA 93407 USA; 7 FibroGen Inc., 409 Illinois Street, San Francisco, CA 94158, USA; 8 Current address: Richmond Chemical Corporation, 2010 Midwest Rd. Oakbrook, IL 60523, USA

## Abstract

**Background:**

Collagens require the hydroxylation of proline (Pro) residues in their triple-helical domain repeating sequence Xaa-Pro-Gly to function properly as a main structural component of the extracellular matrix in animals at physiologically relevant conditions. The regioselective proline hydroxylation is catalyzed by a specific prolyl 4-hydroxylase (P4H) as a posttranslational processing step.

**Results:**

A recombinant human collagen type I α-1 (rCIα1) with high percentage of hydroxylated prolines (Hyp) was produced in transgenic maize seeds when co-expressed with both the α- and β- subunits of a recombinant human P4H (rP4H). Germ-specific expression of rCIα1 using maize globulin-1 gene promoter resulted in an average yield of 12 mg/kg seed for the full-length rCIα1 in seeds without co-expression of rP4H and 4 mg/kg seed for the rCIα1 (rCIα1-OH) in seeds with co-expression of rP4H. High-resolution mass spectrometry (HRMS) analysis revealed that nearly half of the collagenous repeating triplets in rCIα1 isolated from rP4H co-expressing maize line had the Pro residues changed to Hyp residues. The HRMS analysis determined the Hyp content of maize-derived rCIα1-OH as 18.11%, which is comparable to the Hyp level of yeast-derived rCIα1-OH (17.47%) and the native human CIa1 (14.59%), respectively. The increased Hyp percentage was correlated with a markedly enhanced thermal stability of maize-derived rCIα1-OH when compared to the non-hydroxylated rCIα1.

**Conclusions:**

This work shows that maize has potential to produce adequately modified exogenous proteins with mammalian-like post-translational modifications that may be require for their use as pharmaceutical and industrial products.

## Background

Collagen is the most abundant protein found in animals. It has been used widely for industrial and medical applications such as drug delivery and tissue engineering [1,2]. Human type I collagen is the most abundant collagen type in the human body and is also one of the most studied collagen types. It is a heterotrimer composed of two α1 (CIα1) and one α2 (CIα2) chains with the helical region composed by a repeating composition of Xaa-Yaa-Gly, where X and Y are typically proline (Pro) and hydroxyproline (Hyp) [3]. Collagens used commercially are traditionally extracted from animal tissues. These products contain different types of collagen and may be contaminated with potential immunogenic and infective agents considered hazardous to human health. Thus, recombinant technology has been developed to produce high quality and animal derived contaminant-free collagens. Recombinant collagens have been produced in mammalian cells [4], insect cell cultures [5], yeast [6], and plant cell culture [2,7].

Transgenic plant systems have advantages over other recombinant production systems in terms of lower cost, higher capacity, lower infective agents/toxins contamination risk, and inexpensive storage capability facilitating processing [8,9]. The production of plant derived recombinant collagen I α-1 (rCIα1) was reported in 2000 using tobacco [10] and tobacco cell culture [2]. The rCIα1 was also expressed in transgenic maize seed [11,12] and barley [13].

A challenge for producing rCIα1 in non-mammalian expression systems such as transgenic plants is the resulting low regioselective hydroxyproline content that makes the product unstable at physiologically relevant temperatures. In humans the 4-hydroxyproline residues synthesized by prolyl 4-hydroxylases (P4Hs) as a posttranslational modification increase the stability of the collagen triple helix structure [14]. The stability of the collagen is increased with the presence of the hydroxyproline primarily through stereoelectronic effects [15]. On the other hand, the hydroxyproline content for the rCIα1 is almost zero in transgenic tobacco [10], or very low in transgenic maize [11] when rCIα1 is not co-expressed with P4H. Since the insect, microbial and plant endogenous P4Hs are not able to achieve the same level of hydroxylation of rCIα1 as present in the human CIα1 chain, the co-expression with collagen of a recombinant animal P4H (rP4H) is necessary to increase the hydroxyproline content of the rCIα1 to deliver a stable product. In tobacco, co-expression of P4H with an α subunit from *C. elegans *and a β subunit from mouse [16] or a recombinant human P4H [17] led to increased hydroxyproline levels of the rCIα1. Similar results were seen in tobacco cell culture [2]. However, the tobacco-derived collagen still had lower Hyp content compared to native human CIα1 making this product unsuitable for use in many applications.

In this study, we generated transgenic maize lines expressing the human rCIα1 gene alone or lines co-expressing both rCIα1 and rP4H genes. Using high-resolution mass spectrometry (HRMS) analysis, we measured the percentages of Hyp and Pro residues in the rCIα1 protein extracted from transgenic maize seeds as well as the actual positions of hydoxylated prolines. We also performed in vitro pepsin treatment at different temperatures to compare the thermal stabilities of maize-derived hydroxylated or non-hydroxylated rCIα1 proteins. Here, we report for the first time that by co-expressing rP4H genes, maize can produce rCIα1 with a hydroxyproline content comparable to native human type I collagen. This achievement provides further confirmation that maize seeds can be used to produce exogenous proteins that require mammalian-like posttranslational modifications for use in specific applications.

## Results

### Generation of maize lines expressing rCIα1 with and without rP4H co-expression in seeds

**Figure 1:**
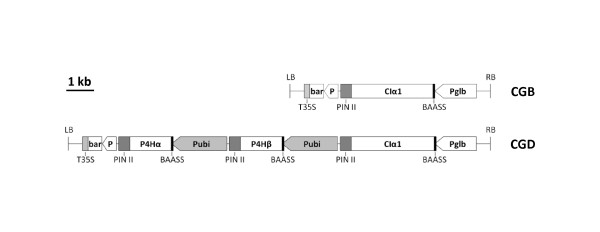
**A schematic representation of the two constructs used in this study**. LB, left border of *Agrobacterium *T-DNA; T35S, CaMV 35S terminator; bar, bialaphos resistant coding sequence; P, CaMV 35S promoter; PIN II, potato protease inhibitor II gene terminator; CIα1, human collagen I α1 chain coding sequence; BAASS, barley alpha amylase signal sequence; P4Hα, prolyl 4-hydroxylase α subunit; P4Hβ, prolyl 4-hydroxylase β subunit; Pglb, maize globulin-1 promoter; Pubi, maize ubiquitin promoter; RB, right border of *Agrobacterium *T-DNA.

The constructs used in this study are shown in Figure 1. The CGB construct carries a gene encoding a recombinant full-length human collagen type I, rCIα1, and the CGD construct carries the rCIα1 gene and both α and β subunits of recombinant human prolyl 4-hydroxylase, rP4Hα and rP4Hβ. The rCIα1 gene was partially maize codon-optimized and its expression was driven by a maize embryo specific globulin-1 promoter (Pglb, [18]). A barley alpha amylase signal sequence (BAASS, [19]) was used as a substitute for the human CIα1 signal peptide (UniProtKB/Swiss-Prot: P02452 [1-22]). The combination of embryo specific promoter and the BAASS has demonstrated high expression of foreign proteins in maize seed [20-22]. The rCIα1 gene lacks the N-propeptide but contains the telopeptide sequences both at the N and C terminal regions. A 29 amino acid bacteriophage T4 fibritin foldon peptide sequence [23] was fused at the C-terminus to the rCIα1 replacing the C-propeptide. The foldon, as the native C-propeptide, facilitates the rCIα1 triple-helical assembly and enhances its stability [23]. To avoid undesired DNA rearrangement caused by using identical sequences (such as using same promoters for multiple gene expression in a single construct), we chose to use the maize ubiquitin promoter (Pubi, [24]) to drive the expression of α and β subunits of rP4H. It was shown previously that there is a preferential accumulation of recombinant protein in germ tissue using the ubiquitin promoter [25].

Both constructs were introduced into maize Hi II germplasm using immature embryo via an *Agrobacterium*-based transformation system. Twelve independent transgenic events for CGB and 21 events for CGD, respectively, were recovered and brought to maturity in the greenhouse using pollen donors from an elite inbred. Initial transgene expression screens were conducted on both callus and T_1 _seeds using an enzyme-linked immunosorbent assay (ELISA) to detect the expression of rCIα1, and α and β subunits of rP4H. T_2 _seeds from events with the highest transgene expressions were produced by self pollination.

For T_1 _seed analysis, seeds from multiple plants derived from each event were analysed. In depth molecular and biochemical characterizations of rCIα1 described in this study were performed on T_2 _seeds from one selected CGB and CGD event, respectively. Individual seeds of the transgenic events were analyzed by polymerase chain reaction (PCR) to separate transgene positive seeds from negative ones. Positive seeds were pooled and analyzed by ELISA for the expression of the rCIα1. Negative null segregant seeds were used as controls.

The average expression level of rCIα1 measured by ELISA in event CGB was 1.86 ± 1.26 mg/g of total soluble protein (TSP) or 12.14 ± 8.06 mg/kg of dry seed weight (DSW). The highest rCIα1 content measured to date from a single CGB seed was 3.54 mg/g TSP or 25.11 mg/kg DSW. The average expression level of the rCIα1 in event CGD was about four times lower than that of in event CGB, which was 0.58 ± 0.26 mg/g TSP or 4.40 ± 2.09 mg/kg DSW. The highest rCIα1 expression in single CGD seed was 0.92 mg/g TSP or 7.54 mg/kg DSW.

**Figure 2:**
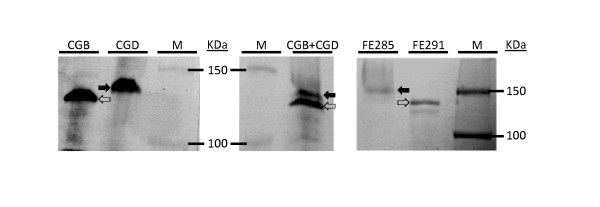
**Analysis of electrophoretic mobility difference of rCIα1 in the CGB and CGD line**. Equal volumes of total protein extracts from seeds of CGB, CGD (10 × concentrated by volume) and mixture of CGB+CGD extracts were loaded on the gel. The rCIα1 from CGB and CGD lines were detected by western blot using anti-foldon antibody. *Pichia*-derived rCIα1 (FE291) and rCIα1-OH (FE285) were included as controls and detected by Coomassie Brilliant Blue staining. Open arrows, rCIα1 from CGB or FE291; solid arrows, OH-rCIα1 from CGD or FE285. M, molecular weight marker.

Figure 2 shows the detection of rCIα1 in the total protein extracts from CGB and CGD seeds. Because of the low expression level of rCIα1 in CGD seeds, we concentrated the extract using an Amicon Ultra-15 Centrifugal Filter Unit with Ultracel-30 membrane (cat # UFC903008, Millipore) before loading on the gel. Figure 2 shows that rCIα1 could be detected from both CGB and CGD protein samples using anti-foldon antibody. No cross-reacting band at a similar position could be detected in non-transgenic maize seeds (data not shown). It was observed that the CGB rCIα1 migrated faster than its CGD counterpart (Figure 2, open arrow in Lane CGB vs solid arrow in Lane CGD), suggesting different electrophoretic mobility for these two proteins. To exclude that the observed protein migration difference was due to lane shifts during electrophoresis, we mixed the TSPs from both CGB and CGD before loading on the gel. Lane CGB+CGD of Figure 2 shows there are two distinct major bands that cross-reacted with anti-foldon antibody. This result indicates that the rCIα1 proteins derived from maize CGB and CGD events have different electrophoretic mobility, with CGD rCIα1 moves slower than CGB rCIα1. The altered electrophoretic mobility may reflect the increase in molecular weight of rCIα1 that due partially to increased numbers of hydroxylated proline in rCIα1 from CGD event, which is also co-expressing the rP4H genes. The difference in electrophoretic mobility can also been seen in *Pichia*-derived rCIα1 (Figure 2, FE291) and hydroxylated rCIα1 (Figure 2, FE285).

**Figure 3:**
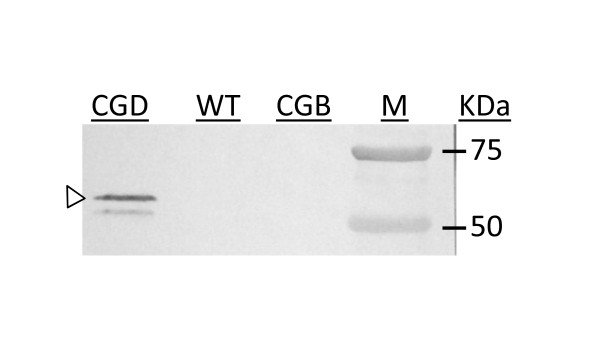
**Western blot analysis of the rP4Hβ using anti-P4Hβ antibody**. Equal volume of total protein extraction from seeds of CGB, CGD and non-transgenic control maize (WT) extracts was loaded on the gel. Open triangle, rP4Hβ. M: molecular weight marker.

The expression of the β subunit of rP4H in the CGD seeds was verified by Western Blotting with an anti P4Hβ monoclonal antibody (Figure 3). A main band at ~60 kD (open triangle, Figure 3) was detected in CGD, but not in CGB and non-transgenic wild type maize control, as expected. A weak secondary band detected in CGD is likely due to cross-reactivity of other forms of rP4Hβ in maize. The detection of α subunit of P4H was performed in transgenic callus but not in seeds (data not shown).

To determine whether rCIα1 can also be detected in tissues other than seeds, we performed both protein and transcript analyses of rCIα1 in CGB and CGD plants. Maize leaf samples from 5 different development stages were collected. Total RNA and proteins were prepared from these tissues and subjected to Reverse Transcriptase PCR (RT-PCR) and ELISA, respectively. No detectable rCIα1 transcript and protein could be observed in these samples (data not shown), suggesting that the rCIα1 is not expressed in leaf tissue in both lines as expected.

### Co-expression of rP4H increases the hydroxylation of rCIα1

To examine the percentage and positions of the prolines that were hydroxylated by the co-expression of rP4H in the CGD event, we carried out proteomics analysis of gel purified rCIα1 using liquid chromatography tandem mass spectrometry (LC-MS/MS) on the Linear Ion Trap Orbitrap (LTQ Orbitrap) Mass Spectrometer, a high resolution mass spectrometry (HRMS). The HRMS not only can verify the amino acid sequence of the rCIα1, but also can identify the positions of hydroxylated proline residues (Hyp). In addition to maize-derived rCIα1 proteins from CGB and CGD, we also included three control samples: gel isolated CIα1 fragment from human collagen (cat # 234138, CalBiochem Inc), *Pichia*-derived rCIα1 (isolated from strain FE291 that does not co-express rP4H) and *Pichia*-derived hydroxylated rCIα1 (isolated from strain FE285 that co-express rP4H) (FibroGen).

**Figure 4:**
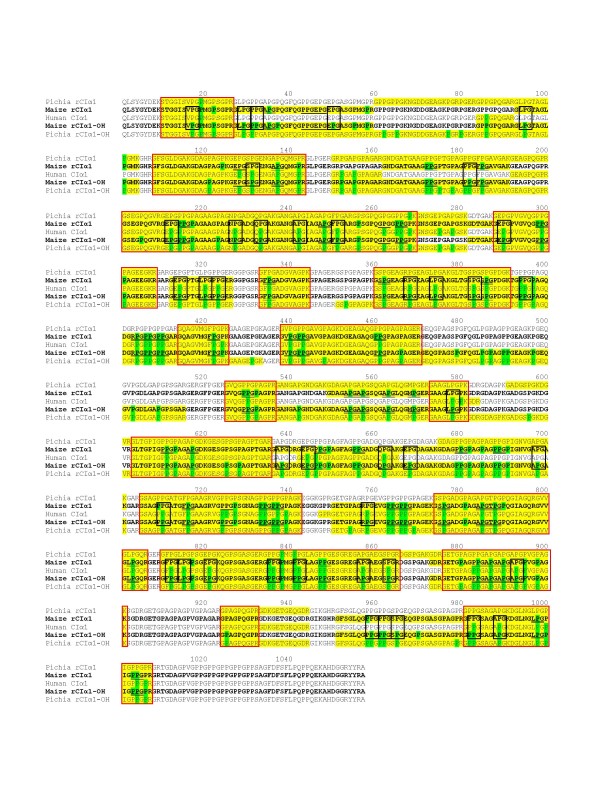
**LC-MS/MS analysis of the rCIα1**. Full length peptide sequences of 1057 amino acid are listed. Pichia rCIα1, *Pichia*-derived rCIα1 from strain FE291; Maize rCIα1, maize-derived rCIα1 from line CGB; Human CIα1, gel-isolated CIα1 fragment from commercial collagen (CalBiochem Inc); Maize rCIα1-OH, maize-derived rCIα1 from line CGD; Pichia rCIα1-OH, *Pichia*-derived rCIα1 from strain FE285. Yellow-highlighted letters: amino acid sequences identified by the Orbitrap; green-highlighted letters: Hyp residues identified by the Orbitrap; red boxes: peptide regions identified in all five samples by the Orbitrap. Black boxes: collagenous triplets Xaa-Pro-Gly with Pro changed to Hyp in Maize rCIα1-OH but not in Maize rCIα1; single underlines: triplets with Pro unchanged in both maize lines; double underlines: triplets with Pro changed to Hyp in both maize lines.

Results are summarized in Figure 4 and Table 1. The protein sequence coverage by the HRMS (yellow highlighted sequences in Figure 4) on the five samples ranged from 58.66% (human CIα1) to 85.81% (*Pichia *CIα1-OH). To compare the percentages and positions of Hyp in each sample, we chose the peptide regions in all five CIα1 proteins (475 AA) that were covered by the HRMS (red boxes in Figure 4). The common peptide regions represent 44.94% of the full-length CIα1 sequence (1057 AA). A total of 114 Pro and Hyp out of 475 total amino acids (24.00%) were identified by the HRMS in all samples (Figure 4).

**Table 1:** Summary of HRMS analysis on five CIα1 (1057 AA^1^) samples from maize, *Pichia *and human

	Pichia CIα1 (FE291)	Maize CIα1 (CGB)	Human CIα1	Maize CIα1-OH (CGD)	Pichia CIα1-OH (FE285)
**Peptides identified by HRMS (highlighted in yellow, Figure 4)**					
Total # AA	680	752	620	818	907
Percent HRMS coverage	64.33%	71.14%	58.66%	77.39%	85.81%
					
**Peptide regions identified in all five C1α1 (475 AA) by HRMS (red boxes, Figure 4)**					
Total # HYP identified by HRMS, in green	2	28	71	86	83
Percent HYP identified by HRMS	0.42%	5.89%	14.95%	18.11%	17.47%
					
% HYP (by AA analysis)	N/A	1.23%^2^	10.8%^3^	N/A	11.54%^2^

^1^sequences presents in all five samples

^2^from reference [11]

^3^from reference [28]

For two maize-derived CIα1 samples, a total of 28 and 86 Hyp were identified from CGB and CGD (green highlighted amino acids in Figure 4), respectively, representing a Hyp percentage of 5.89% and 18.11%, respectively, for these two lines (Table 1). This result indicates that the co-expression of rP4H in maize can greatly enhance the hydroxylation of prolines on collagen molecules. The increased number of Hyp in rCIα1 from CGD samples may partially contributed to the increased molecular weight and thereby decreased the migration rate (Figure 2).

Because rP4H catalyzes hydroxylation of Pro residues in the Yaa position of the Xaa-Yaa-Gly triplets within collagen strands [26], we further compared the Pro residues on all Xaa-Pro-Gly triplets in both maize CGB and CGD lines. HRMS analysis identified 752 AA (71.14%) from maize-rCIα1 (CGB) and 818 AA (77.39%) from maize rCIα1-OH (CGD) as shown in Table 1 and Figure 4 (bold and yellow highlighted letters). Among these HRMS identified AA, we chose 652 AA that were shared for both CGB and CGD. We further identified a total of 90 sets of collagenous triplets within the 652 AA. Among the 90 sets of triplets, 44 sets (48.9%) have the Pro residues changed to Hyp (double underlined triplets in Figure 4) in both CGB and CGD lines; 5 sets (5.6%) have the Pro unchanged (single underlined triplets in Figure 4) in both lines. On the other hand, 41 sets of triplets (45.6%) have the Pro residues changed to Hyp (black boxes in Figure 4) only in rCIα1 isolated from CGD, indicating that nearly half of the collagenous triplets were posttranslationally modified by the co-expression of rP4H genes in CGD maize line.

Seventy-one Hyp residues out of 475 AA by HRMS (14.95%) were identified in human CIα1 control sample (Table 1). For *Pichia *samples, while only two Hyp residues (0.42%) were found in non-hydroxylated CIα1 (FE291), 83 Hyp residues (17.47%) were found in hydroxylated CIα1 (FE285), indicating that the co-expression of P4H in *Pichia *had also dramatically increased proline hydroxylation in collagen (Table 1).

### Co-expression of rP4H enhances the thermal stability of rCIα1

**Figure 5:**
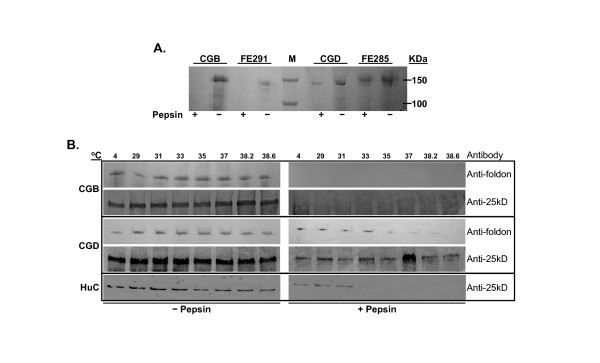
**Thermal stability analysis of the rCIα1 from maize, *Pichia *and human**. (A) Western blot results of the maize-derived rCIα1 (CGB), rCIα1-OH (CGD), *Pichia*-derived rCIα1 (FE291), and rCIα1-OH (FE285) after 4°C incubation and pepsin treatment, using anti-foldon antibody. (B) Western blot results of the maize-derived rCIα1 (CGB), rCIα1-OH (CGD), and human CIα1 (HuC) after heat treatments under various temperatures and pepsin treatments as indicated, using both anti-foldon and anti-25 kD collagen antibody. M: molecular weight marker.

To further characterize the maize-derived rCIα1 and rCIα1-OH, we carried out thermal stability analysis using pepsin digestion at 10°C for 15 minutes after heat treatment of protein samples at 4°C or temperatures ranged from 29 to 38.6°C for 6 minutes. The proteolytic resistance of maize-derived collagens were compared with that of the native human collagen and the recombinant collagen from *Pichia pastoris*. Figure 5A is a Western Blot results showing the proteolytic resistance of the collagens after 4°C heat treatment using anti-foldon antibody. Both non-hydroxylated rCIα1 from maize (CGB) and non-hydroxylated rCIα1 from *Pichia *(FE291) were not detected after pepsin treatment. By contrast, the hydroxylated rCIα1 could be detected from both maize (CGD) and hydroxylated-CIα1 *Pichia *(FE285) samples, suggesting that the pepsin digestion resistance of these collagens was associated with the higher percentage of Hyp residues.

The thermal stability of rCIα1 was further characterized by the determination of melting temperature (Tm) using Western analysis. Two different antibodies, anti-foldon and anti-25 kD collagen, were used. In our hands, anti-foldon antibody gave results with less non-specific cross-reactive background bands, while anti-25 kD antibody appeared to be more sensitive. Because the native human CIα1 can only be detected with anti-25 kD antibody, we used both antibodies in this study. In the experiments shown in Figure 5B, maize seeds TSP from CGB and CGD were extracted and concentrated as described above. The quantities of maize-derived rCIα1 were estimated by ELISA. Approximately 50-100 ng/reaction of rCIα1 from CGB and CGD were used for pepsin treatment. As a control, commercial human collagen (2 μg/reaction) was spiked into TSP extracted from non-transgenic maize seed for pepsin treatment. As can be seen in Figure 5B, both maize-derived rCIα1 (CGB) and rCIα1-OH (CGD) were as stable as the human collagen control at all temperatures tested in the absence of pepsin. When digested with pepsin, the maize-derived non-hydroxylated rCIα1 (CGB) was degraded after the heat treatment at temperatures as low as 4°C. On the other hand, the hydroxylated rCIα1-OH (CGD) could still be detected after temperature treatment as high as 35°C when using anti-foldon antibody, and 38.6°C when using anti-25 kD antibody. The difference in Tm results was likely due to the sensitivity and epitope recognition sites of two types of antibodies. Interestingly, the control native human collagen could only withstand the digestion upto temperature treatment around 31°C. This observation is in fact in agreement with the HRMS analysis of the collagens described in Table 1 and Figure 4. Because the maize-derived rCIα1-OH has higher Hyp percentage (18.11%) than that from human collagen control (14.95%), it is expected that the increased Hyp residues could help to increase the thermal stability of the collagen molecules.

## Discussion

The production of plant-derived recombinant collagens have been reported in tobacco leaves, barley cell culture and seeds, as well as maize seeds as summarized in Table 2. Previous tobacco-derived rCIα1 studies showed that different combinations of recombinant human collagens (i.e. rCIα1, rCIα2, and N-propeptide free rCIα1) were used to improve the production of homotrimeric or heterotrimeric recombinant human type I collagen [10,16,17,27]. In a recent paper, Stein *et al *[17] achieved a high expression level of 200 mg/kg fresh leaves by expressing the collagens under a *Chrysanthemum* rbcS1 promoter and vacuolar-targeting signal sequence. Early work with tobacco-derived collagens had very low levels of Hyp (0.53%, [10]). With co-expression of *C. elegans *P4Hα/Mouse P4Hβ [16] or the human rP4Hα/β [17], Hyp levels were increased to 8.41% and 7.55%, respectively. However, this enhanced Hyp level in tobacco is still lower than that of native human collagen CIα1, which is reported as 10.8% by amino acid analysis [28] or around 15% by the HRMS analysis (this work).

**Table 2:** Summary of plant derived recombinant human collagen I α 1

Expression system	Collagen			rP4H	Hydroxylation content (%)	Reference
				
	Regulatory sequences	Gene	Yield			
Tobacco Leaf	P35S (constitutive) + PR-protein SP	proα1(I) ΔNproα1(I)	30 mg/kg powdered plants	N/A	0.53	10
Tobacco Leaf	P35S (constitutive) + PR-protein SP	ΔNproα1(I)	N/A	N/A	N/A	27
Tobacco Leaf	L3 + PR-protein SP	ΔNproα1(I)	0.5-1 mg/kg leaf material	P1287 + Native SP + C. elegans P4Hα/Mouse P4Hβ	8.41	16
Tobacco	rbcS1 (constitutive) + vacuole or apoplast targeting SPs	proα1(I)/proα2(I)	200 mg/kg fresh leaves	P35S (constitutive) + vacuole or apoplast targeting SPs + Human P4Hα/β	7.55	17
Barley P1 cell	Ubi (constitutive) + At chitinase SP + HDEL (ER retention)	proα1(I)	2-9 μg/L cell culture	N/A	N/A	7
Barley Seed	GluB1 (endosperm specific) + At chitinase SP + HDEL (ER retention)	CIα1 45 kD	Below detectable level (CIα1) 45 mg/kg seed (45 kD)	N/A	N/A (CIα1) 2.8 (45 kD)	13
Maize Seed	globulin-1 (embryo specific) + barley α-amylase SP	44 kD	20 mg/kg seed	N/A	2.01	29
Maize Seed	globulin-1 (embryo specific) + barley α-amylase SP	CIα1	3 mg/kg seed	N/A	1.23	11
Maize Seed	globulin-1 (embryo specific) + barley α-amylase SP	CIα1 44 kD	15.9 mg/kg germ (CIα1) 49.6 mg/kg germ (44 kD)	N/A	N/A	12
Maize Seed	globulin-1 (embryo specific) + barley α-amylase SP	CIα1	12 mg/kg seed (CIα1) 4 mg/kg seed (CIα1-OH)	Pubi (constitutive) + Barley α-amylase SP + Human P4Hα/β	18.11	This study

proα1(I): human type I procollagen α 1 chain

ΔNproα1(I): human type I procollagen α 1 chain lacking N-propeptide

ΔNCproα1(I): human type I procollagen α 1 chain lacking N-propeptide and C-propeptide

CIα1: sequence information is not clear

44 kD: 44 kD fragment of CIα1

45 kD: 45 kD fragment of CIα1

Both the full length and a smaller fragment (45 kD) of rCIα1 were produced in barley cell culture [7] and barley seeds [13]. The barley-derived 45 kD collagen has 2.8% of Hyp content when produced in seeds without co-expression of rP4H genes [13].

Previous work on fractionation, purification and characterization of maize-derived full length and a smaller fragment (44 kD) of collagen suggested that an accumulation level of about 3 mg/kg (for the full length) and of 20 mg/kg (for the 44 kD) of DSW, respectively [11,29]. A similar maize line accumulating the full length rCIα1 producing maize line (CGB) was used in this study. In our case, the collagen yield of the rCIα1 accumulating line without P4H co-expression averages 12 mg/kg DSW, while the rCIα1 accumulating line with P4H co-expression (CGD) is about 4 mg/kg. The Hyp percentage in rCIα1 protein of CGB was reported as 1.23% using total amino acid composition (AAC) analysis [11]; however, it was measured at 5.89% by using HRMS analysis in our study. Similarly, the Hyp percentage in human CIα1 was reported as 10.8% using AAC analysis [28]. In our HRMS analysis, the Hyp for human CIα1 measured around 15%. It is not clear why Hyp percentages of CIα1 proteins measured uniformly higher in HRMS analysis than that of in AAC analysis. This discrepancy is likely due to the different degrees of resolution of these two very different methodologies. Because the concentrations of rCIα1 and rCIα1-OH obtained from maize seeds were too low to be measured by AAC analysis in our study, we were not able to obtain AAC analysis results for comparison.

P4H is an enzyme that regioselectively modifies the Pro residues in collagenous triplets Xaa-Pro-Gly [30,31] in the ER as a posttranslational modification. Compared to the *Pichia *recombinant protein production system, maize can produce hydroxylated rCIα1 with a comparable Hyp percentage (Table 1, 18.11% in maize CGD vs 17.47% in *Pichia *FE285). Interestingly, rCIα1 produced in maize seems to have a higher base-level Hyp percentage when compared to rCIα1 isolated from *Pichia *with no rP4H co-expression (Table 1, 5.89% in maize CGB vs 0.42% in *Pichia *FE291). Small numbers of proline at both Xaa and Yaa positions got hydroxylated in CGB maize line without the co-expression of P4H (data not shown). It is likely that the rCIα1 produced in maize is also a substrate for plant endogenous P4Hs with lower efficiency [30].

Conversely, the expression of human rP4H in maize may also catalyze hydroxylation of Pro residues in any plant endogenous proteins with collagenous domains. We checked amino acid sequences of three abundant seed storage proteins (19 kD and 22 kD α-zein, and 27 kD γ-zein) in maize and did not find any collagenous triplets (X. Xu, unpublished). Therefore we do not expect any Pro to Hyp modifications on these seed storage protein in the rP4H expressing CGD line. In fact, the Hyp-only AAC analysis on both CGB and CGD seeds showed no differences in Hyp contents (X. Xu, unpublished). However, because both α and β subunits of rP4H were under the control of the constitutive ubiquitin promoter, it is possible that any of the collagenous triplet domains on proteins in plant cells can be modified by rP4H in such transgenic lines. It may be desirable in the future to restrict rP4H expression to seed tissue only using seed specific promoters.

Using HRMS to analyze posttranslational modification has obvious advantages such as low protein quantity requirement, free of contaminating proteins in samples and reading accuracy. However, it does not give 100% coverage. In this study the peptide coverage ranged from 58.66% to 85.81%.

Because posttranslational modification is a continuous process in the cells, the collagen isolated from the seeds represents a population of protein molecules, i.e., the proline hydroxylation may vary from one collagen molecule to another. In fact, we have performed multiple HRMS measurements on samples extracted from same batch of seeds. We found that while positions of Pro to Hyp modification may vary between measurements, the overall Hyp content remained constant between these samples.

The thermal stability tests in this report showed that maize-derived rCIα1-OH could still be detected after pepsin digestion followed by heat treatment as high as 35°C (using anti-foldon antibody) and 38.6°C (using anti-25 kD antibody). Commercial human collagen control undergoing the same treatment could only withstand up to 31°C temperatures. Stein *et al *[17] reported that the melting temperatures for their tobacco-derived collagen heterotrimer and human skin collagen samples were around 39°C. High melting temperature of plant-derived collagen could potentially be useful for certain industrial application where higher melting temperature is desired, for example, biomaterials for tissue engineering [32,33].

We recovered the maize-derived rCIα1 from the seed total soluble proteins using a previously described protocol [11]. Because collagens are acid soluble proteins, the extraction buffer used had a pH of 1.7. Unlike Zhang *et al*. [11], we did not perform extensive purification for rCIα1 before gel electrophoresis and Western blot analysis. When treating such acidic rCIα1 solutions under high temperature as we normally do before loading protein gels, we were unable to detect them in Western blot, suggesting that the combination of acidic buffer and high temperature could be detrimental to collagen integrity. Therefore in this study, all maize-derived rCIα1 samples in acidic solutions were not boiled prior to Western blot analysis to avoid collagen degradation.

It is interesting to note that both maize- and *Pichia*-derived non-hydroxylated rCIα1 were completely digested by pepsin at 10°C after the temperature treatment of samples at 4°C in our study (Figure 5A). This result is different from what was reported in barley [7] and maize [11], in which plant- and *Pichia*-derived rCIα1 were still detectable after the heat treatment of 26-27°C. This could be attributed to the different pepsin treatment protocols used in the experiments. For example, the pepsin experiments reported by Zhang *et al*[11] were conducted under pH 7, with a 15 minutes heat treatment followed by 150 μg/mL pepsin digestion at 4°C for 16-18 hr. Ritala *et al*[7] conducted the heat treatment for 6 min before subjecting the samples to 150 μg/mL pepsin digestion at 10°C for 30 min under an acidic condition. Our conditions were similar to Ritala *et al *except that we used 200 μg/mL pepsin for 15 min under pH 1.7. Because pepsin functions best in acidic environment, our pepsin digestion under low pH is likely leading to the degradation of non-hydroxylated rCIα1 even at 4°C. Another explanation could be the quantity of the collagen substrate used in different experiments. We estimated that approximately 50-100 ng/reaction of unpurified rCIα1 from CGB seeds were subjected to pepsin digestion in our study. However, Zhang *et al* used about 600 - 700 ng purified rCIα1 per reaction in their study [11]. The quantity of collagen for pepsin digestion in Ritala *et al*[7] was not specified.

We have demonstrated for the first time that mammalian-like hydroxylation of human rCIα1 can be achieved in transgenic maize co-expressed with a human rP4H. The Hyp content in maize-derived hydroxylated rCIα1 is comparable to that of the native human version, leading to a similar thermal stability of the product. The current expression levels of collagen reported here are too low for large scale production, as desired accumulation level of recombinant proteins for commercial production is estimated between 250 to 1000 mg/kg grain [34,35]. Further improvement of recombinant protein production in plants can be achieved by optimization of gene expression including using more effective regulatory elements and protein targeting/retention sequences, as well as using conventional breeding program to select high expression lines over generations [34,36].

## Conclusions

In this study we have shown that properly hydroxylated recombinant human collagen I alpha 1 (rCIα1) can be produced in maize seed. By co-expressing recombinant human prolyl 4-hydroxylases (rP4Hs), we have successfully produced rCIα1 containing Hyp residue levels that are comparable to native human CIα1. The increased Hyp content is associated with increased thermal stability in maize-derived rCIα1. Application of high-resolution mass spectrometry (HRMS) allowed us to measure hydroxylated prolines at specific amino acid positions in different samples. Our findings indicate that maize seed can be used as a system to produce recombinant proteins requiring mammalian-like posttranslational modifications.

## Methods

### Vector construction

Human collagen type I α 1 (CIα1) coding sequence together with its original N- and C-telopeptides sequences (UniProtKB/Swiss-Prot: P02452) were optimized by Aptagen LLC (Jacobus, PA) for expression in maize. The optimized CIα1 sequence was fused with a 29 amino acids bacteriophage foldon peptide sequence [23] at the C-terminus to produce a protein with 1086 amino acids. Two constructs (Figure 1) were made to produce either recombinant CIα1 (rCIα1) only (CGB), or both rCIα1 and recombinant human prolyl-4-hydroxylase (rP4H, CGD). The rCIα1 gene was regulated by a maize embryo-specific promoter, globulin-1 [18], with a 3'-terminator from potato protease inhibitor II (pin II) gene. Genes encoding two subunits of rP4H (rP4Hα and rP4Hβ) were regulated by a maize constitutive promoter (ubiquitin promoter) and the potato pin II gene terminator. All three gene coding sequences (rCIα1, rP4Hα, and rP4Hβ) in the two constructs were translationally fused with a barley alpha amylase signal sequence (BAASS, [19]) at the 5' end. The phosphinothricin acetyl transferase (bar) gene driven by the cauliflower mosaic virus (CaMV) 35S promoter was adopted in both constructs to be a marker for the transgenic callus selection. It confers resistance to the herbicide glufosinate ammonium (bialaphos) [37-39].

### Production of transgenic plants

Constructs CGB and CGD were introduced into immature embryos of Hi II maize genotype [40] via an *Agrobacterium*-based transformation system [41]. Briefly, maize immature embryos were infected by *Agrobacterium *strain EHA101 [42] containing the above described vectors and selected on 3 mg/L bialaphos. Regeneration of transgenic plants from the callus was as previously described [20]. Seedlings were transplanted into soil in the greenhouse and allowed to flower and produce seed through hand-pollinations. Seed increases for multiple events from CGB and CGD were conducted in greenhouse and nursery trials. T_2_ transgenic maize seeds were used for further analysis in this study.

### PCR analysis of transgenic plants

Total genomic DNA was isolated by Cetyl Trimethyl Ammonium Bromide (CTAB) method [43] from maize leaf or seed. The presence of transgenes rCIα1, rP4Hα and rP4Hβ were detected by polymerase chain reaction (PCR). A typical PCR reaction consists 100 ng of genomic DNA, 0.8 mM of dNTPs, 2 mM of MgCl_2_, Taq DNA polymerase buffer and 0.5 U Taq DNA polymerase (Bioline USA Inc, Taunton, MA) in a final volume of 25 μL. PCR was performed at the following condition for 35 cycles: 30 s denaturation at 94°C, 30 s annealing at 60°C, and 45 s extension at 70°C. Primers for amplifying rCIα1 are x7-05 (5'-ACCAGATGGGCCGCTCTCACCTTT-3') and x7-06 (5'-TTCCCTGGTGCCGTTGGAGCTA-3'); for rP4Hα are x7-17 (5'-ATCTCGGCGTCGCTGATGAT-3') and x7-18 (5'-GTGGTCCGAGCTGGAGAACC-3'); and for rP4Hβ are x7-13 (5'-ATGAAGAACACCTCCTCCCTCTG-3') and x7-14 (5'-TCACAGCTCGTCCTTCACGG-3'). PCR products were analyzed in 1% agarose gel. The expected sizes of PCR products are 1308 bp (rCIα1), 745 bp (rP4Hα) and 1531 bp (rP4Hβ), respectively. Gel was stained by ethidium bromide (0.5 μg/ml) for 20 min. The products size was determined by 1 kb DNA Ladder (cat # N3232S, New England Biolabs).

### Protein extraction

Total soluble protein (TSP) from maize seeds was extracted using an acidic buffer described by [11] for collagen preparation. Maize seeds were ground in a coffee grinder (Mr. Coffee) for 1 min. For rCIα1 extraction, extraction buffer (0.1 M phosphoric acid, 0.15 M sodium chloride, pH 1.7) was added in to the seed powder at the ratio of 1:10 (w/v). For rP4H extraction, extraction buffer [25 mM sodium phosphate (pH 6.6), 100 mM sodium chloride, 0.1% Triton X-100 (v/v), 1 mM EDTA, 10 μg/mL leupeptin, and 0.1 mM serine protease inhibitor Perfabloc SC (Fluka)] was added into the seed powder at the ratio of 1:10 (w/v). The mixture was incubated in a shaker incubator (250 rpm, 37°C) for 0.5 hour for rCIα1 and one hour for rP4H. The mixtures were then centrifuged at 13,000 rpm for 10 min at room temperature in a bench top centrifuge. The supernatants were transferred to clean tubes for further analysis. Some protein samples were concentrated by Amicon Ultra-15 Centrifugal Filter Unit with Ultracel-30 membrane (cat # UFC903008, Millipore) followed the product instruction. In short, 15 mL of total seed protein extraction was loaded into the filter device, centrifuged at 3000 × g for approximately 2-3 hours at 4°C. Concentrated samples were recovered by withdrawing with a pipettor. The concentration level was measured by the volume and could be adjusted by the control of the centrifugation time.

### ELISA

A competitive ELISA procedure developed by FibroGen and described by Zhang *et al*. [29] was used with minor modifications. Briefly, ELISA plates (cat # 3590, Corning) were coated overnight at 4°C with 5 ng per well of heat-denatured (65 ± 5°C for 30 minutes) non-hydroxylated rCIα1 from *Pichia pastoris *(FE301,[7]) with phosphate buffer saline (PBS, cat # 21-040-CV, Mediatech). After washing with washing buffer (10 mM PBS, 0.05% Tween 20, pH 7.0), the plates were blocked with 2% dry milk in 100 mM PBS for 1 hour at room temperature. After 3 × washings with washing buffer, heat-denatured samples and standard (FE301) in assay buffer (100 mM PBS, 0.05% Tween 20, 1% dry milk, pH 7.0) were added to the plates. The primary antibody, rabbit polyclonal anti-25 kDa CIα1 (CA725, FibroGen), was added immediately at a 1:4000 dilution in the assay buffer. After 1 hour incubation at room temperature, plates were washed 3 × with washing buffer. The goat-anti-rabbit IgG (H+L) HRP conjugate (cat # 81-6120, Zymed) was added at a 1:5000 dilution in the assay buffer followed by incubation at room temperature for 1 hour. After 3 × washings with washing buffer, 100 μL/well of Sure Blue TMB substrate solution (cat # 52-00-01, Kirkegarrd & Perry Laboratories) were added. The plated were then read at 620 nm on a microplate reader (KC4, Biotek) after incubation at room temperature for 30 minutes.

### Western blotting

Forty microliters of protein extract from maize seed were mixed with 8 μL of Laemmli sample buffer (cat # 161-0737, Bio-Rad) and then loaded onto a 4-15% polyacrylamide SDS-PAGE gel (cat # 161-1158, Bio-Rad). To avoid protein degradation in the combination of acidic pH and high temperature (X. Xu, unpublished), the step of sample boiling prior to loading was omitted. The proteins separated on the gel were transferred to a 0.45 μm nitrocellulose membrane using Bio-Rad Semidry Transblotting apparatus according to the manufacturer's instructions. Membranes were incubated in blocking buffer (138 mM sodium chloride, 2.7 mM potassium chloride, pH 7.4, 0.1% Tween-20, 5% dry milk powder) for 1 hour at room temperature on a rotary shaker. The membrane was then incubated for 1 hour in blocking buffer with 1:1000 dilution of anti-foldon antibody (rabbit anti-sera with 0.01% sodium azide) for the rCIα1, and with 1:1000 dilution of anti-P4Hβ antibody (cat # 63-164, ICN Biomedicals) for the rP4Hβ. After washing with washing buffer (138 mM sodium chloride, 2.7 mM potassium chloride, pH 7.4, 0.05% Tween-20) 4 times (5 min each wash), the membrane was then incubated for 1 hour in blocking buffer with 1:5000 dilution of HRP-Goat anti-rabbit IgG (H+L) secondary antibody (cat # 62-6120, Zymed) for the rCIα1, and with 1:5000 dilution of HRP-Goat anti-mouse IgG (H+L) secondary antibody (cat # 62-6520, Zymed) for the P4Hβ. After washing the membrane with washing buffer 4 × 5 min, the excess buffer was then drained off and the membrane transferred into a clean container. Bands appeared after incubation with horseradish peroxidase substrate, 3,3', 5,5'-tetramethylbenzidine (cat # T0565, Sigma) within 10 minutes.

### High-resolution mass spectrometry (HRMS) analysis

To prepare maize-derived rCIα1, 10 μg of total soluble proteins extracted from seeds was separated on the 4-15% polyacrylamide SDS-PAGE gel followed by Bio-Safe Coomassie Stain (cat # 161-0786, Bio-Rad). For purified collagen control samples, three micrograms of each of *Pichia*-derived non-hydroxylated collagen (FE291), *Pichia*-derived hydroxylated collagen (FE285), and human collagen (cat # 234138, CalBiochem Inc.) were loaded on the gel. After electrophoresis, collagen bands were excised from the gels and sent to the Proteomics & Mass Spectrometry Facility at Donald Danforth Plant Science Center, St. Louis, MO for analysis. The samples were automatically digested with trypsin performed by MultiProbe II protein digester (PerkinElmer) in a temperature-controlled enclosed environment. After digestion, samples were run by LC-MS/MS on the Linear Ion Trap Orbitrap (LTQ-Velos Orbitrap, ThermoFisher Scientific). For post-translational modification analysis, the numbers of Hyp and Pro from each sample were counted and compared.

### Thermal stability analysis

The melting temperature (Tm) of CIα1 samples was determined by pepsin digestion after heat treatment [23]. Twenty-five microliters of total soluble protein extracted from CGB and CGD maize seed was subjected to heat treatment in a Thermocycler machine (Biometra GmbH, Germany) at 4°C, or at temperatures ranged from 29°C to 38.6°C for 6 min. For positive controls, 1.4 μg of *Pichia*-derived rCIα1 in hydroxylated (FE285) and non-hydroxylated (FE291) forms, and human collagen were also treated. After heat treatment, all protein samples were then incubated at 10°C with or without pepsin (0.2 mg/mL final concentration, cat # P6887, Sigma) for 15 min. Digestion results were analyzed by western blotting using anti foldon and anti 25 kDa collagen antibodies.

## Authors' contributions

XX carried out all the molecular analysis on T_2 _transgenic seeds and drafted the manuscript. QG assisted with XX for protein analysis. RC made constructs for maize transformation and conducted the molecular analysis. KP carried out the biochemical analysis in maize plants. JH, JB and KW conceived the study and review the paper. KW designed the experiment and edited the paper. All authors read and approved this final manuscript.
